# Immune deficiency phenotypes of *Il2rg*, *Rag2* or *Il2rg/Rag2* double knockout rats; establishment of human leukemia xenograft models

**DOI:** 10.1186/s42826-024-00231-5

**Published:** 2024-12-27

**Authors:** Joo-Il Kim, Hyun-Jin Lim, Euna Kwon, Tomoji Mashimo, Byeong-Cheol Kang

**Affiliations:** 1 Department of Experimental Animal Research, Biomedical Research Institute, Seoul National Univ. Hospital, Seoul, Korea; 2 Graduate School of Translational Medicine, Seoul National University College of Medicine, 101 Daehak-ro, Jongno-gu, Seoul, 03080 Republic of Korea; 3 Division of Animal Genetics, Laboratory Animal Research Center, Institute of Medical Science, The University of Tokyo, Minato-ku, Tokyo, Japan; 4 Institue of Laboratory Animal Resources, Seoul National University, Seoul, Korea

**Keywords:** SCID rat, Phenotype, Immunodeficiency, Human leukemia xenograft model

## Abstract

**Background:**

Genetically immunodeficient mice lacking *Il2rg* and *Rag2* genes have been widely utilized in the field of biomedical research. However, immunodeficient rats, which offer the advantage of larger size, have not been as extensively used to date. Recently, Severe Combined Immunodeficiency (SCID) rats were generated using CRISPR/Cas9 system, targeting *Il2rg* and *Rag2* in National BioResource Project in Japan. We imported and investigated more detailed phenotypes of wild-type (WT) *Il2rg* knockout (KO)*, Rag2* KO and *Il2rg*/*Rag2* KO rats for 20 weeks.

**Results:**

During experiments, *Il2rg* KO*, Rag2* KO and *Il2rg*/*Rag2* KO rats showed decreased white blood cells and systemic lymphopenia, with reduced CD4+, CD8+ T cells and CD161+ NK cells. Additionally, all KO strains exhibited reduced relative spleen weights, hypoplasia of the germinal center in the white pulp, and atrophy with the disappearance of the boundary between the cortex and medulla in the thymus, compared to WT rats. Furthermore, we established human acute lymphoblastic leukemia xenograft rat model by intravenously injecting 5.0 × 10^6^ cells/kg of NALM6 cells into *Il2rg/Rag2* KO rats.

**Conclusions:**

These findings indicate that *Il2rg* KO, *Rag2* KO, and *Il2rg/Rag2* KO rats exhibited SCID phenotypes, suggesting their potential application as immunodeficient animal models for tumor xenograft studies.

## Background

Immunodeficient mice play a crucial role in biomedical fields for in vivo studies [1, 2]. Non-obese diabetic (NOD) and Cg-*Prkdc*^*scid*^* IL2rg*^*tm1Wjl*^/SzJ (NSG) mice are the most widely-used strains, lacked T, B and natural killer (NK) cells [3, 4]. However, the small size of the mouse poses limitations in sample collection or surgery, and there are constraints in terms of the concentration of cells that can be injected and tumor size. Moreover, in non-clinical toxicity studies, when human-derived stem cells are intravenously injected into mice, they predominantly distribute in the lungs and liver, potentially leading to pulmonary embolism or intravenous thrombosis [5–7]. This can complicate toxicity prediction and distribution evaluations. In contrast, rats, which are ten times larger than mice, allow for greater sample collection and easier surgical procedures, representing a viable alternative [8, 9]. However, immunodeficient rats are not widely utilized in current research fields.

Previously, He et al*.* generated *Rag1/Rag2/Il2rg* knockout (KO) rats and Miyasaka et al*.* successfully established F344-*Il2rg*^*em1Iexas*^ (*Il2rg* KO)*,* F344-*Rag2*^*em1Iexas*^ (*Rag2* KO) and F344-*Il2rg/Rag2*^*em1Iexas*^ (*Il2rg*/*Rag2* KO, FRG) rats using CRISPR/Cas9 system and analyzed immunodeficient phenotype in 2019 and 2022 [10, 11].

The *Il2rg* gene encodes the common gamma chain, an essential component of multiple cytokine receptors involved in lymphocyte development and function [12–14], while *Rag2* is indispensable for V(D)J recombination during T and B cell maturation [15–17]. Importantly, the choice of the F344 background strain offers advantages in terms of genetic stability, reproducibility, and well-characterized physiological parameters [18].

In this study, we imported the rats from the National BioResource Project Japan, and conducted more detailed phenotypic analysis including clinical signs, body weight measurements, food/water intake, hematological and serum biochemical analyses, urine analysis, coagulation test, organ weights, and histopathological examinations from 4 to 20-week-old.

To demonstrate the practical application of SCID rats, we successfully established a xenograft model of acute lymphoblastic leukemia (ALL) model by intravenously injecting human-derived leukemia cells, NALM6, and confirmed robust engraftment using in-vivo imaging system (IVIS).

This study aims to provide detailed phenotypes of F344-*Il2rg* KO, *Rag2* KO, and *Il2rg/Rag2* KO rats, shedding light on their overall health status, physiological parameters, and histopathological features in lymphoid organs correlated with the severe lymphopenia caused by *Il2rg* and *Rag2* KO. Additionally, it demonstrates that the FRG rat can be a valuable model for assessing the efficacy of anticancer drugs across a variety of human cancers.

## Methods

### Animals

Heterozygote F344-*Il2rg*^*em1Iexas*^ (*Il2rg* KO)*,* F344-*Rag2*^*em1Iexas*^ (*Rag2* KO) and F344-*Il2rg/Rag2*^*em1Iexas*^ (*Il2rg*/*Rag2* KO, FRG) rats were purchased from the National BioResource Project—Rat, Kyoto University (Kyoto, Japan). After breeding and genotyping, homozygous *Il2rg* KO, *Rag2* KO and *Il2rg*/*Rag2* KO rats were used in phenotypic analysis with their wild-type (WT) littermates as a control group. Animals of the same gender and genotype were housed under a 12 h/12 h light/dark cycle, a temperature of 22 ± 2 °C, and a humidity of 40–60%. They had free access to food (Teklad Certified. Irradiated Global 18% Protein Rodent Diet 2918C, Envigo, USA) and sterilized water. Animals were maintained in a SPF facility accredited by AAALAC International (#001169) in accordance with Guide for the Care and Use of Laboratory Animals 8th edition, National Research Council. All experiments were approved by the Institutional Animal Care and Use Committee of Seoul National University Hospital (SNUH-IACUC No. 19-0011).

### Physiological phenotypes measurement of animals

We measured physiological phenotypes of *Il2rg* KO, *Rag2* KO, *Il2rg/Rag2* KO and wild type when they were 4 weeks of age and terminated at 20 weeks. During this period, clinical signs were monitored daily, body weights, food and water intake were measured once a week.

### Hematology, flow cytometry, serum biochemistry and coagulation test

At the end of the study, whole blood was collected from the inferior vena cava in a tube containing K_2_EDTA (BD Microtainer) for the total and differential counting of white blood cells (WBC) and red blood cells (RBC), platelets as well as determination of hemoglobin, mean corpuscular volume (MCV), mean corpuscular hemoglobin (MCH), and mean corpuscular hemoglobin concentration (MCHC) using ADVIA 2120i (Siemens Healthcare, Tarrytown, NY, USA). For flow cytometry, whole blood of additional WT, *Il2rg* KO*, Rag2* KO *and Il2rg/Rag2* KO rats (*n* = 3 per strain) were obtained and removed RBC using RBC lysis buffer (Invitrogen, USA) and cell counting, 1 × 10^7^ cells were stained with the following antibodies (APC anti-rat CD3; clone 1F4; CAT#201414, PE/Cyanine7 anti-rat CD8a; clone OX-8, CAT#201716, FITC-anti-rat CD4; Clone W3/25, CAT#201505, PE anti-rat CD161; clone 3.2.3, CAT#205604 and all antibodies were from BioLegend USA). All samples were incubated for 30 min at 4 °C, and analyzed using a LSR II Flow Cytometer (BD biosciences, USA).

Serum biochemistry analysis was performed using the Hitachi 7070 (Hitachi, Tokyo, Japan) to measure blood urea nitrogen, total cholesterol, total protein, albumin, aspartate transaminase, alkaline phosphatase, alanine transaminase, gamma-glutamyl transferase, creatinine, triglyceride, glucose, albumin/globulin ratio, potassium, chloride, sodium and phosphorus, and coagulation tests were carried out using the CA-620 (Sysmex, Kobe, Japan) for prothrombin time (PT) and activated partial thromboplastin time (aPTT).

### Histopathologic analysis

At 20 weeks old, all rats were euthanized and organs were observed macroscopically. Liver, spleen, kidney, brain, lung, heart, adrenal glands, testis or uterus, thymus, thyroids and pituitary were weighed and fixed in 10% neutral buffered formalin, while eyes and testis were preserved in Davidson and Bouin’s solution, respectively. The fixed organs were embedded in paraffin wax and stained with hematoxylin and eosin (H&E) after they were sectioned into 4–6 μm thick slices. Histopathological analyses of the liver, spleen, pancreas, kidney, brain, lung, heart, adrenal glands, gastrointestinal tract, femur, sternum, nasal cavity, ovary, and testis were performed.

### Establishment of acute lymphoblastic leukemia (NALM6) xenograft rat model

Human acute lymphoblastic leukemia cell line NALM6 was purchased from the American Type Culture Collection (ATCC) and maintained in RPMI1640 medium (Welgene, Republic of Korea) supplemented with 10% fetal bovine serum (FBS) (Welgene, Republic of Korea) and 1% Penicillin–Streptomycin (Gibco, U.S.A). Rats (*n* = 3) were received 5.0 × 10^6^ cells/kg of luciferase-labeled NALM-6 cells via the tail vein. After injection, animals were monitored clinical signs daily and were evaluated using in vivo imaging system (IVIS spectrum, PerkinElmer, U.S.A) on days 7, 14, 18, 24 and 30 after NALM6 injection. Animals were euthanized under deep anesthesia when body weight decreased by more than 20% or hind limb paralysis due to leukemic burden.

### Statistical analysis

All data are expressed as the mean ± SD. Statistical analysis was carried out using one-way ANOVA, followed by multiple comparison procedure with Dunnet test using SPSS software version 25.0 (SPSS Inc., Chicago, IL, USA). *P* values less than 0.05 was considered as statistically significant.

## Results

### *Il2rg* KO, *Rag2* KO, *Il2rg/Rag2* KO rats showed normal physiological parameters

**Fig. 1:**
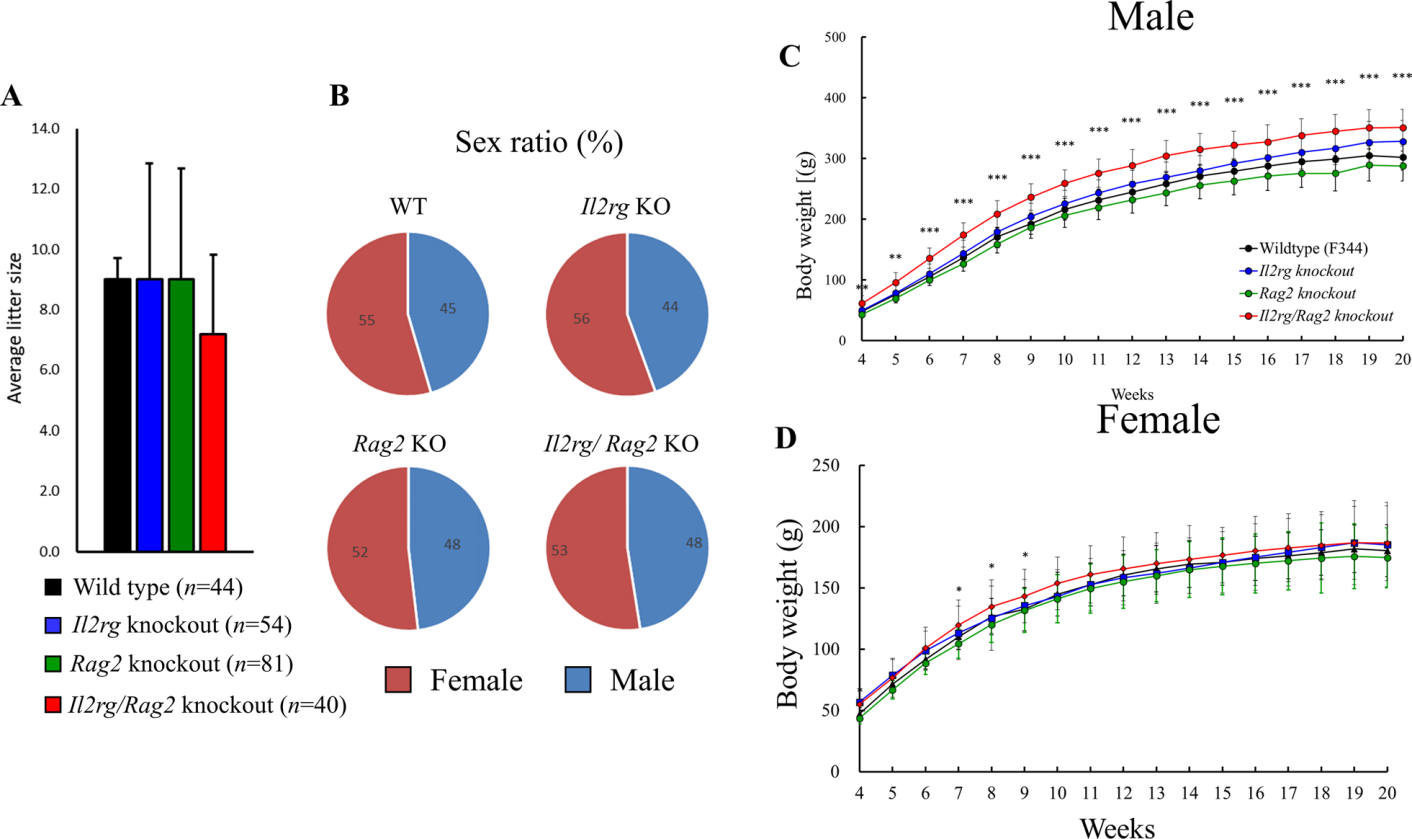
*Il2rg* KO*, Rag2* KO *and Il2rg/Rag2* KO rats showed normal birth rate, sex ratio and body weights for 20 weeks. **A**, **B** There were no significant differences observed in average birth rates and sex ratios. Body weights of *IL2rg/Rag2* KO rats increased significantly compared to WT rats. **C** In males, this difference was observed from 4 to 20 weeks of age, while in **D** females, it was observed from 7 to 9 weeks of age. Data are presented as mean ± standard deviation compared to WT. *p < 0.05, **p < 0.01 and ***p < 0.001

Previously, Miyasaka et al*.,* generated SCID rats using CRISPR/Cas9 system with mutations in *Il2rg* and *Rag2* genes (10). These rats showed immunodeficient phenotypes, characterized by decreased WBC counts and hypoplasia in thymus and spleen. To further analyze the phenotypes of these rats, we monitored their development and physiological parameters from fetus to adulthood (*n* = 10 per strain). There were no statistically significant changes in the number of birth and sex ratio in WT (5 deliveries, male; n = 20, female n = 24), *Il2rg* KO (7 deliveries, male, n = 24, female, n = 30), *Rag2* KO (13 deliveries, male, n = 39, female, n = 42) and *Il2rg/Rag2* KO (7 deliveries, male, n = 19, female, n = 21) (Fig. 1A, B). Throughout 20 weeks, the body weights of *IL2rg/Rag2* KO rats increased significantly compared to WT rats, with significant increases observed from 4 to 20 weeks of age in males and from 7 to 9 weeks of age in females (Fig. 1C, D). This difference in body weights may be attributed to a lower number of pups born to *IL2rg/Rag2* knockout rats, which could result in larger individual weights. However, consistent with the prior study by Miyasaka et al., we confirmed that there was no effect on rat development. These results suggested that there were no effects of *Il2rg* and *Rag2* gene on delivery rate, gender determination and physiological development.

### Decreased WBC and lymphocytes counts with loss of CD3+ cells in peripheral blood in *Il2rg* KO, *Rag2* KO and *Il2rg/Rag2* KO rats

**Fig. 2:**
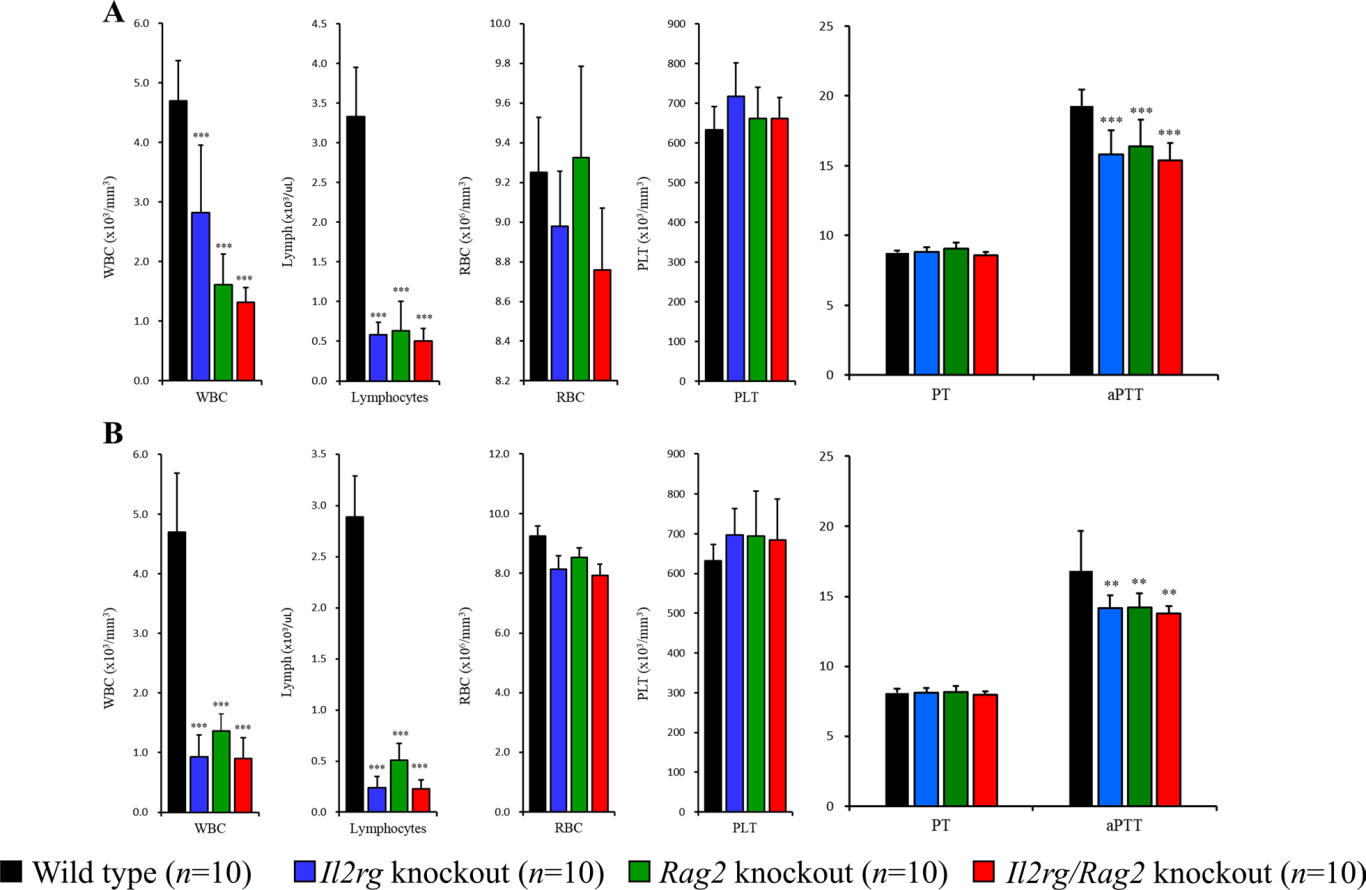
Hematologic analysis of peripheral blood of SCID rats. Decreased WBC and lymphocytes counts in *Il2rg* KO, *Rag2* KO, *Il2rg/Rag2* KO rats both **A** male and **B** female compared to wild-type (WT). Red blood cell and platelet counts remained unchanged, while the activated partial thromboplastin time (aPTT) level was observed to be lower than in SCID rats than WT during coagulation analysis, but it was confirmed to be within the normal range. Data are presented as mean ± standard deviation compared to WT. *p < 0.05, **p < 0.01 and ***p < 0.001

At the 20 weeks of age, rats were euthanized and performed hematology analysis using peripheral blood. Total WBC counts of *Il2rg* KO (Male; 2.82 ± 1.13, Female; 0.93 ± 0.37 × 10^3^/ul), *Rag2* KO (Male; 1.61 ± 0.51, Female; 1.36 ± 0.28 × 10^3^/ul), *Il2rg/Rag2* KO (Male; 1.32 ± 0.25, Female; 0.91 ± 0.34 × 10^3^/ul) rats were significantly reduced compared to those of age and gender-matching WT rats (Male; 4.70 ± 0.67, Female; 3.70 ± 0.99 × 10^3^/ul) (Fig. 2). When determining the detailed profiles of WBC, differential counting showed that lymphocyte counts were significantly decreased in *Il2rg* KO (0.24 ± 0.11 × 10^3^/ul), *Rag2* KO (0.51 ± 0.16 × 10^3^/ul), *Il2rg/Rag2* KO (0.23 ± 0.08 × 10^3^/ul) rats compared to WT rats (2.89 ± 0.40 × 10^3^/ul) and all values were statistically significant (*p* < 0.001). On the other hand, there were no differences in neutrophils, basophils, and monocytes, RBC counts, platelets, and reticulocytes (Supplementary Table 1).

**Fig. 3:**
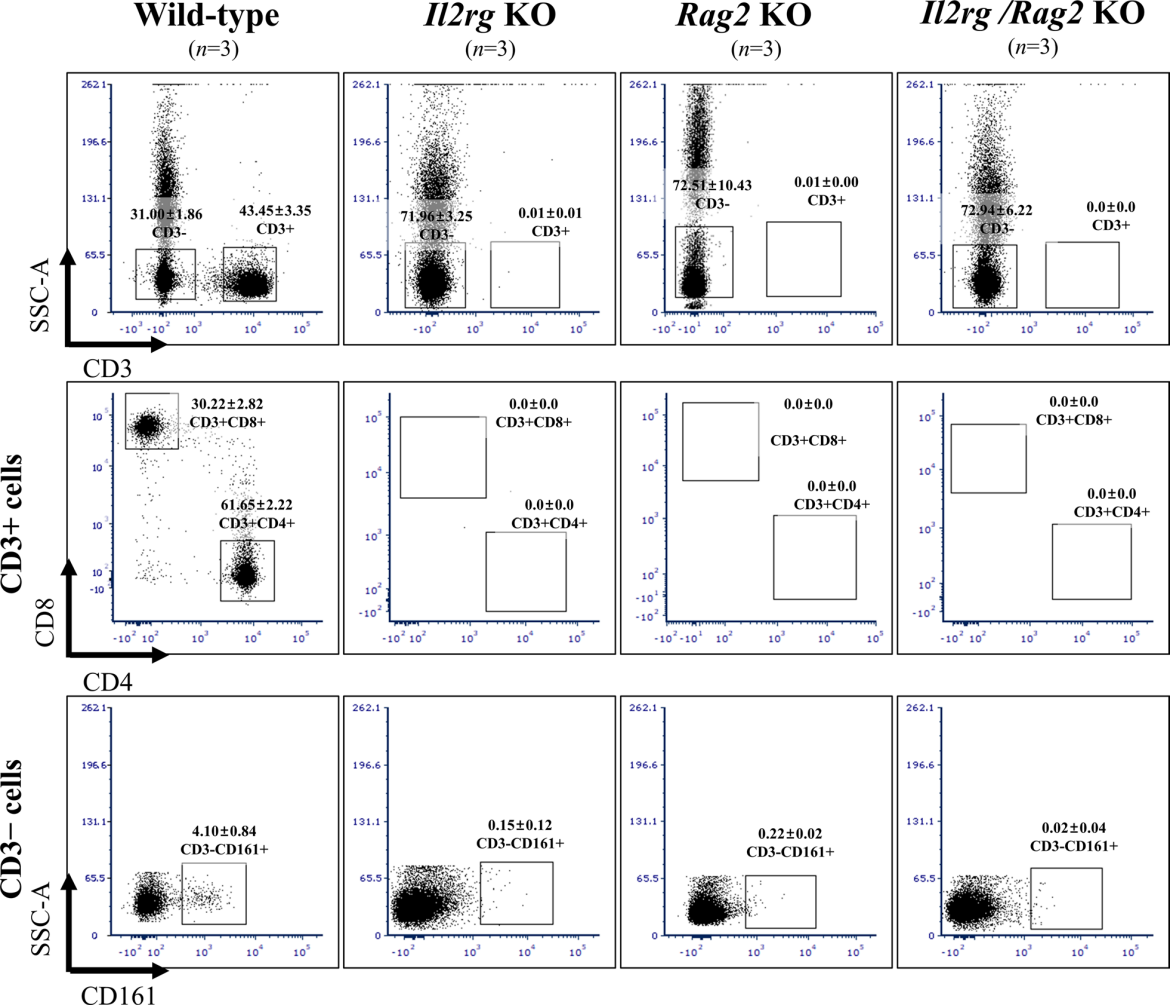
Flow cytometry analysis of lymphocyte population, isolated from the peripheral blood. *Il2rg* KO, *Rag2* KO, *Il2rg/Rag2* KO rats showed decreased CD3+ CD4+, CD3+  CD8 T cells and CD3-CD161+ NK cells compared to age-matched WT rats. Data are presented as mean ± standard deviation

To examine changes in lymphocyte populations, we conducted flow cytometry analysis on peripheral blood samples. We observed a significant reduction in CD3 + T cells in *Il2rg* KO (0.01 ± 0.01%), *Rag2* KO (0.01 ± 0.00%), and *Il2rg/Rag2* KO (0.00 ± 0.00%) rats compared to WT rats (43.45 ± 3.35%), as well as in CD3 + CD8 + (WT; 30.22 ± 2.82%) and CD3 + CD4 + (61.65 ± 2.22%) T cells. Additionally, CD3-CD161 + NK cells decreased in *Il2rg* KO (0.15 ± 0.12%), *Rag2* KO (0.22 ± 0.02%), and *Il2rg/Rag2* KO (0.02 ± 0.04%) rats compared to WT rats (4.10 ± 0.84%) (Fig. 3).

**Fig. 4:**
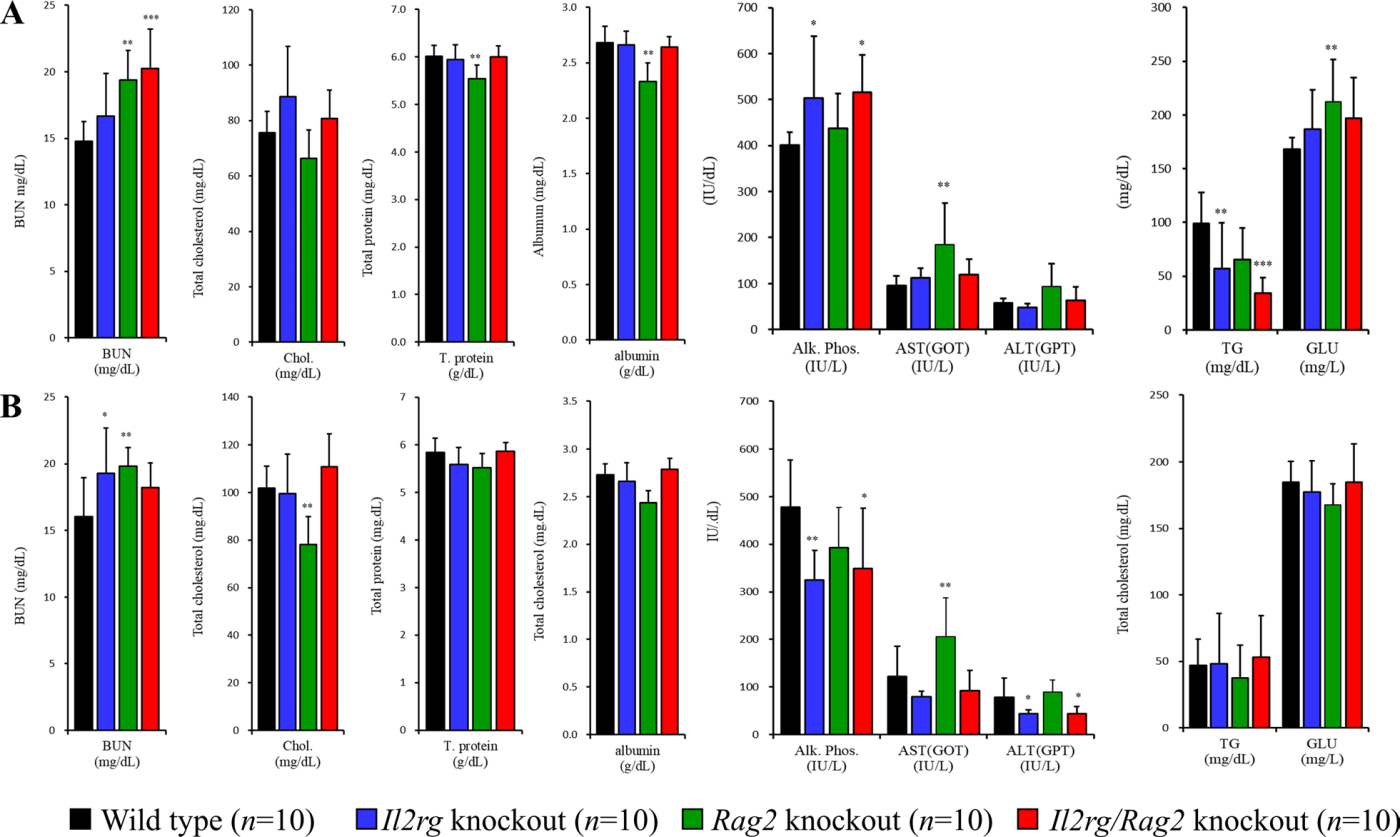
Serum biochemical showed normal ranges in *Il2rg* KO, *Rag2* KO, *Il2rg/Rag2* KO rats. There were significant differences observed in blood urea nitrogen (BUN), total cholesterol (Chol), total protein (T proteins), albumin, ALP, AST, ALT, triglyceride and glucose (GLU) compared to wild-type (WT). But it was confirmed to be within the normal range of F344 background both** A** male and** B** female. Data are presented as mean ± standard deviation. *p < 0.05, **p < 0.01 and ***p < 0.001

Serum chemistry results showed statistical significance in blood urea nitrogen (BUN), total cholesterol (Chol), total protein (T proteins), albumin, Alkaline Phosphatase (ALP), Aspartate Aminotransferase (AST), Alanine transaminase (ALT), triglyceride (TG), and glucose (GLU) levels were compared to those of the wild-type (WT). However, it was confirmed that these levels were within normal range of F344 background (Fig. 4A; male, B; female and Supplementary Table 2).

### Hypoplasia of thymuses and spleens in *Il2rg* KO, *Rag2 *KO and *Il2rg/Rag2* KO rats

**Fig. 5:**
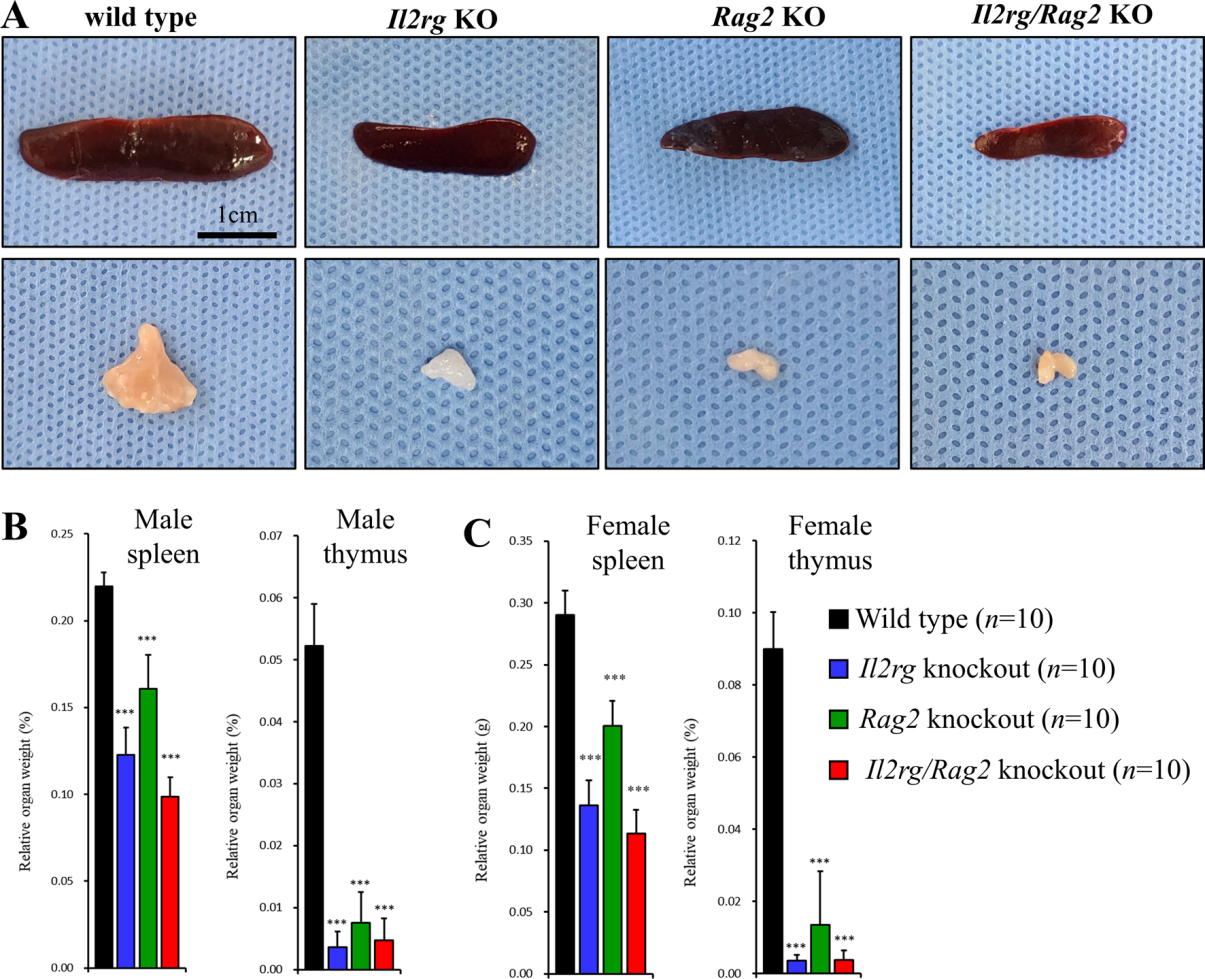
Hypoplasia of thymuses and spleens in *Il2rg* KO*, Rag2* KO*, Il2rg/Rag2* KO rats. **A** Images of spleen and thymus from wild-type (WT), *Il2rg* KO*, Rag2* KO and *Il2rg/Rag2* KO rats. Relative organ weights of spleen and thymus was significantly decreased in both **B** male and **C** female in SCID rats compared to WT. Data are presented as mean ± standard deviation compared to WT. Scale bar indicates 1 cm and all figures share the same scale bar. *p < 0.05, **p < 0.01 and ***p < 0.001

At the end of experiment, all animals were sacrificed at 20 weeks of age and necropsies were performed to measure the organ weights. During necropsy, relative thymus and spleen weights of *Il2rg* KO (male thymus; 0.0036 ± 0.0025%, male spleen; 0.1227 ± 0.0157%, female thymus; 0.0035 ± 0.0016%, female spleen; 0.1362 ± 0.0200%), *Rag2* KO (male thymus; 0.0076 ± 0.0050%, male spleen; 0.1607 ± 0.0197%, female thymus; 0.0134 ± 0.0050%, female spleen; 0.1607 ± 0.0197%), *Il2rg/Rag2* KO (male thymus; 0.0164 ± 0.0134, male spleen; 0.0988 ± 0.0110%, female thymus; 0.0038 ± 0.0026, female spleen; 0.1134 ± 0.0194%) rats were significantly reduced compared to those of age and gender-matching WT rats (male thymus; 0.0522 ± 0.0068%, male spleen; 0.2196 ± 0.0081%, female thymus; 0.0900 ± 0.0102%, female spleen; 0.2900 ± 0.0200%), and all values were statistically significant (Fig. 5) (*p* < 0.001).

### Histopathologic analysis of lymphoid organs in *Il2rg* KO, *Rag2 *KO and *Il2rg/Rag2* KO rats

**Fig. 6:**
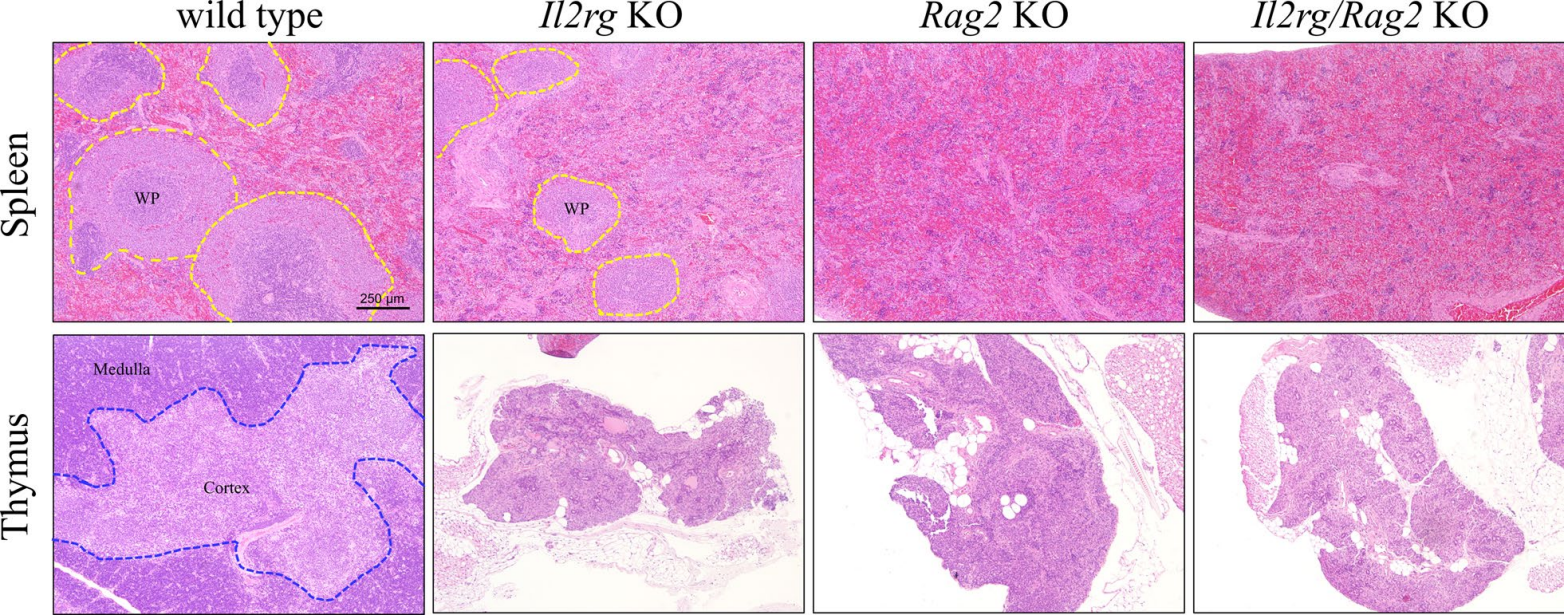
Reduced splenic white pulp and thymic cortex in *Il2rg* KO*, Rag2* KO*, Il2rg/Rag2* KO rats. Performing histopathological analysis of the spleen (upper panel) and thymuses (lower panel) through H&E staining, a reduction in the splenic white pulp (dotted yellow lines) and the disappearance of the boundary (dotted blue lines) between the cortex and medulla of the thymus were observed in three strains. Scale bar indicates 250 μm and all figures share the same scale bar

Histopathological examination of the lymphoid organs including thymus and spleen was performed after H&E staining (Fig. 6). *Il2rg* KO, *Rag2* KO, and *Il2rg/Rag2* KO rats, the distinction between the cortical and medullary regions of thymus disappeared, whereas a clear distinction and structure were observed in WT rats. In the spleen, the white pulp of *Rag2* KO and *Il2rg/Rag2* double KO rats was significantly reduced compared to WT rats. Conversely, *Il2rg* KO rats displayed a normal splenic structure when compared to WT rats; however, the absence of germinal centers within the white pulp was noted, indicating that the conserved formation of the germinal center in spleen is significantly affected by the *Rag2* gene, not the *Il2rg.*

### Successful establishment of human acute lymphoblastic leukemia in *Il2rg/Rag2* KO rats

**Fig. 7:**
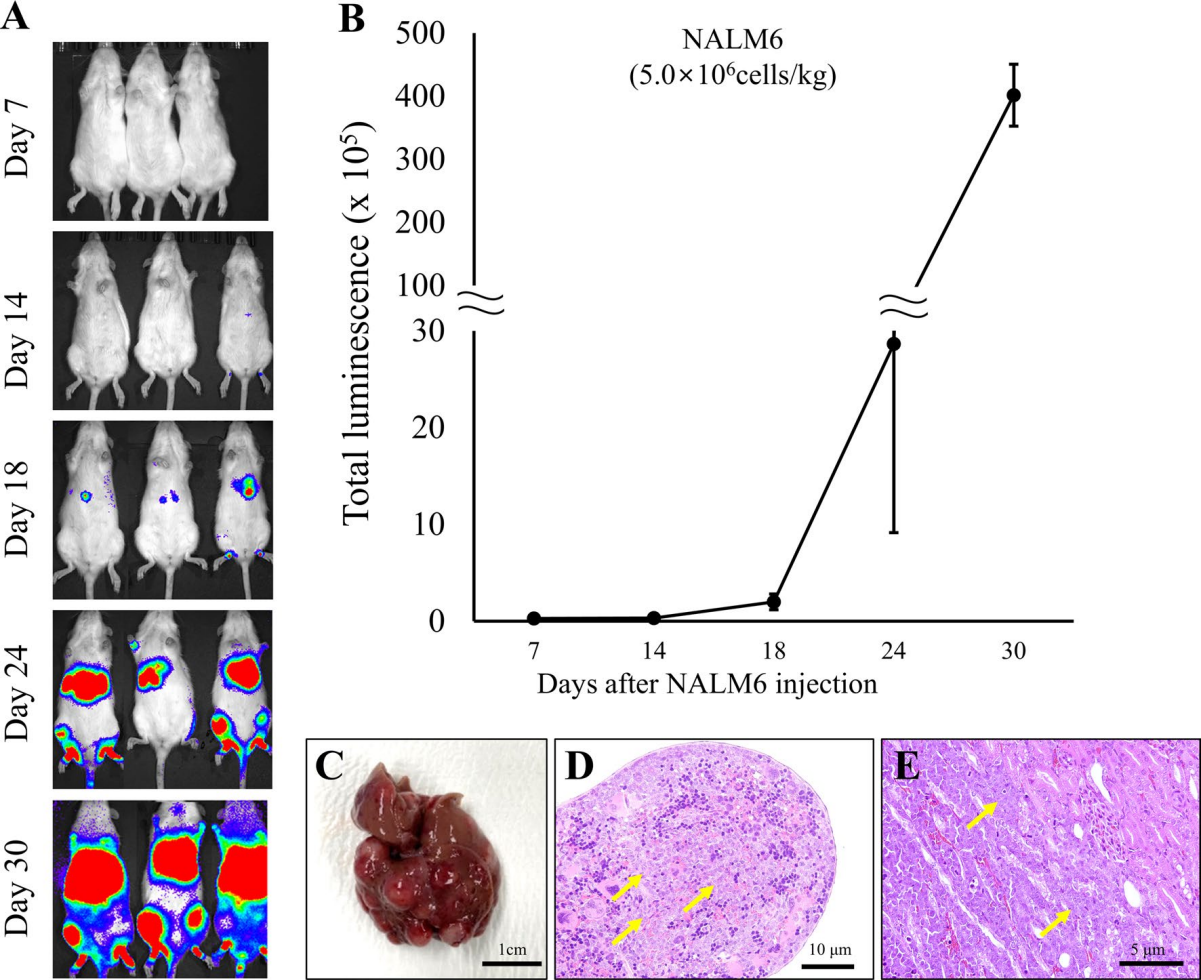
*In-vivo* bioluminescence imaging of NALM6 xenograft rats showed systemic leukemia progression. **A** Bioluminescence imaging was conducted 7, 14, 18, 24 and 30 days after NALM6 injection, and signal was observed starting from day 14, followed by rapid progression. **B** Quantification of bioluminescence. **C** At 35 days after tumor inoculation, autopsy revealed tumor metastasis to the liver. H&E images showed tumor cell infiltration (yellow arrow) in the **D** spleen and **E** kidney. All data are expressed as mean ± standard deviation (SD)

To validate the xenograft model in oncology research, we injected human-derived leukemia cells (NALM6) intravenously into *Il2rg/Rag2* KO rats and analyzed them using IVIS. Bioluminescence (BLI) signals began to be detected in the liver on day 14 and gradually increased over time (Fig. 7A). By day 18, the luminescence intensity had increased to 2.0 × 10^5^ p/s/cm^2^/s, which was higher than the intensity observed on day 14 (0.3 × 10^5^ p/s/cm^2^/s). On day 24, the tumors had progressed to the bone marrow in addition to the liver, leading to a further increase in luminescence intensity (28.6 × 10^5^ p/s/cm^2^/s), representing a 14.3-fold increase compared to day 18. By day 30, the luminescence intensity had increased by 14-fold (401.3 × 10^5^ p/s/cm^2^/s) compared to day 24 (Fig. 7B). The rats exhibited hind limb paralysis and immobility, and they were euthanized on day 35. During autopsy, severe white masses were found diffusely distributed in the liver (Fig. 7C). H&E histopathological analysis confirmed the presence of metastasis in the liver, spleen, kidneys, femur, sternum, and nasal cavity (Fig. 7D, E)

## Discussion

Rat models, distinguished by their larger body size compared to mice, offer promising advantages for utilization in biomedical research. The increased size of rats allows for more precise surgical procedures, enabling researchers to simulate and study complex diseases and therapeutic interventions with greater accuracy. Furthermore, rat models provide an ideal platform for pharmacological studies, toxicology assessments, and the development of novel therapeutic strategies [19]. However, most immunodeficient animals currently used in immunology and cancer studies are mice, making it challenging to overcome the limitations associated with their small size.

In 2010, Mashimo et al. generated X-SCID rats using Zinc-finger nuclease (ZFN) targeting the *Il2rg* gene, and these rats exhibited immunodeficient phenotypes, including hypoplastic thymus, reduced spleen size, and decreased WBC counts. In 2022, Miyasaka et al. generated SCID rats using the CRISPR/Cas9 system to target the *Il2rg* and *Rag2* genes, demonstrating severe immunodeficient phenotypes [10, 20]. Loss of *Il2rg* and *Rag2* significantly affects the development and function of T, B and NK cells due to their crucial roles in the immune system. *Il2rg* is a critical component of the interleukin-2 receptor, which is necessary for T cell proliferation and survival and B cell development. Loss of *Il2rg* leads to impaired development and function of T and B cells, resulting in reduced cell numbers and compromised immune responses [13]. Similarly, *Rag2* is involved in V(D)J recombination, a process essential for generating mature T and B cells [17].

In this study, we characterized the phenotypes of *Il2rg* KO, *Rag2* KO, *Il2rg/Rag2* KO, and their littermates WT rats, performing a more detailed hematological analysis than that presented by Miyasaka et al. in 2022 [10]. Additionally, we conducted phenotypic analysis of *Rag2* single KO rats to examine the impact of each gene on the phenotype. Over the 20-week observation period, we found no changes in sex ratio, litter size and development compared to sex- and age-matched WT, suggesting that *Il2rg* and *Rag2* genes did not affect physiological growth or maturation in F344 background. However, we observed a deficiency of *Il2rg* and *Rag2* that led to decrease in WBC and lymphocyte counts across all strains. Severe reduction in CD3 + T cells was observed in all strains, while CD3-CD161 + NK cells were found to be significantly decreased in *Il2rg/Rag2* KO rats, as confirmed by flow cytometry analysis.

Additionally, splenic and thymic hypoplasia accompanied by histopathological changes were observed, confirming their status as immunodeficient rats. In particular, in *Il2rg* KO mice, it was observed that normal structure of spleen, but the germinal center was significantly decreased in white pulp compared to WT rats as reported previously [21]. All strain of rats exhibited absence of the boundary between the medulla and cortex of the thymus compared to WT rats. This is a phenotype commonly seen in SCID animals and is characteristic of lymphocyte depletion caused by absence of *Il2rg* and *Rag2*.

Rats with knockout of the *Il2rg* and *Rag2* genes exhibit significant immunodeficiency, characterized by a reduction in WBC counts and hypoplasia of the spleen and thymus, which are hallmark features of a SCID phenotype. These features are also observed in *Il2rg* and *Rag2* knockout mice, reflecting the loss of both T and B lymphocytes due to the absence of functional Rag2 (which is crucial for V(D)J recombination) and Il2rg (which is essential for signaling through several interleukin receptors) [22]. Both models share similar characteristics, such as the inability to mount an adaptive immune response, making them invaluable for human xenograft studies.

However, there are notable differences between rat and mouse model. The larger body size of rats offers significant advantages, such as ability to collect larger volumes of blood and tissue samples, better tolerance to surgical procedures, and the capacity to inject higher volumes of cells or therapeutic agent which is particularly useful in preclinical studies. Furthermore, rats generally have a more complex physiology that can more accurately represent human biology, potentially enhancing the translational relevance of research findings to human studies compared to mouse models.

To assess the utility of immunodeficient rat models, we established xenograft rat models by intravenously injecting human acute lymphoblastic leukemia cells (NALM6), observing that tumor cells engrafted slower than previously reported in mice. In contrast to mice, luciferase was readily detectable in the thick-skinned rats, and metastasis to the liver was confirmed through necropsy. These findings indicate that SCID rats can be utilized not only for solid tumor xenografts but also for hematological cancer models, highlighting their potential significance in oncology research.

In this study, we successfully established the first ALL xenograft rat model, which serves as a significant milestone. However, the scope of the current research is limited to this specific ALL model. We aim to expand on this by developing additional tumor models using various hematologic malignancies and solid tumor cell lines. Future studies will focus on evaluating the efficacy and safety of precision therapeutics, including immunotherapy, utilizing these advanced models.

We conducted a comprehensive phenotypic analysis of three strains of SCID rats produced by the Japanese NBRP, employing detailed phenotypic analysis. While all strains exhibited an immunodeficient phenotype, *Il2rg/Rag2* KO rats displayed the lowest levels of CD3-CD161 + NK cells, suggesting their potential utility for further investigations. In addition, their larger size and more robust vascular system compared to mice make them particularly well-suited for cell transplantation experiments, emphasizing their adaptability in such studies.

Furthermore, we established xenograft tumor models by intravenously injecting human acute lymphoblastic leukemia cells and confirming them using IVIS, suggesting their high usefulness in future studies within the field of oncology. This model holds promise not only for anti-cancer drug screenings, but also for mechanistic studies of cancer and potentially for assessing the efficacy and safety of human-derived biopharmaceuticals.

## Conclusions

These findings indicate that *Il2rg* KO, *Rag2* KO, and *Il2rg/Rag2* KO rats exhibited SCID phenotypes, suggesting their potential use as immunodeficient animal models. Additionally, the successful establishment of ALL xenograft models further confirms their applicability for tumor xenograft studies.

## Data Availability

The data and materials supporting the findings of this study are available from the corresponding author upon reasonable request.

## Abbreviations

ALL: Acute lymphoblastic leukemia
ALP: Alkaline Phosphatase
ALT: Alanine transaminase
aPTT: Activated partial thromboplastin time
AST: Aspartate Aminotransferase
ATCC: American Type Culture Collection
BUN: Blood urea nitrogen
Chol: Total cholesterol
FBS: Fetal bovine serum
GLU: Glucose
H&E: Hematoxylin and eosin
IVIS: In-vivo imaging system
KO: Knockout
MCH: Mean corpuscular hemoglobin
MCHC: Mean corpuscular hemoglobin concentration
MCV: Mean corpuscular volume
PT: Prothrombin time
RBC: Red blood cells
TG: Triglyceride
TP: Total protein
WBC: White blood cells
WT: Wild type
ZFN: Zinc-finger nuclease
